# Resistive Switching Behavior of Magnesium Zirconia Nickel Nanorods

**DOI:** 10.3390/ma13122755

**Published:** 2020-06-17

**Authors:** Tzu-Han Su, Ke-Jing Lee, Li-Wen Wang, Yu-Chi Chang, Yeong-Her Wang

**Affiliations:** Institute of Microelectronics, Department of Electrical Engineering, National Cheng-Kung University, Tainan 701, Taiwan; s0964348@hotmail.com (T.-H.S.); Luke.k.j.lee@gmail.com (K.-J.L.); jk220052@gmail.com (L.-W.W.); christina780712@gmail.com (Y.-C.C.)

**Keywords:** bipolar, memory, nanorod, resistive, hydrothermal method

## Abstract

To effectively improve the uniformity of switching behavior in resistive switching devices, this study developed magnesium zirconia nickel (MZN) nanorods grown on ITO electrodes through hydrothermal method. The field emission scanning electron microscope image shows the NR formation. Al/MZN NR/ITO structure exhibits forming-free and bipolar resistive switching behaviors. MZN NRs have relatively higher ON/OFF ratio and better uniformity compared with MZN thin film. The superior properties of MZN NRs can be attributed to its distinct geometry, which leads to the formation of straight and extensible conducting filaments along the direction of MZN NR. The results suggest the possibility of developing sol–gel NR-based resistive memory devices.

## 1. Introduction

Resistive random access memory (RRAM) is one of the strong candidates of next-generation nonvolatile memories (NVM) due to its simple metal/insulator/metal structure, low power consumption, fast switching speed, simple fabrication, and high scalability [1,2,3]. Resistive switching characteristics have been demonstrated in a variety of materials, including binary metal oxides, chalcogenide materials, and perovskite oxides. Perovskite oxides have attracted much attention due to its simple fabrication, low cost, and controllable process [4,5].

ZrO_2_ is a good and well-studied ceramic material that exists in three crystalline phases, namely monoclinic, tetragonal, and cubic phases, at different temperatures. Mg^2+^ ions in Mg:ZrO_2_ with lower valance state cause vacancy in the sub-lattice of oxygen, which improves the performance during volume expansion of the phase transformation. Acetylacetone in Ni (II) acetylacetone possesses two pairs of bidentate ligands (BLs) and offers extremely low polymerization rate, resulting in the smoother surface of MZN films. The smoother surface may have fewer defects and low high-resistance switching (HRS) current. In addition, this strong ligand contributes to stability due to the strong bond between BL and Zr [6,7]. However, local conduction filament in thin films is diverse in each switching, leading to the non-uniform distribution of switching voltages and current distributions which are crucial to the memory array application. Reducing the dimension of devices and achieving a high packing density are also important [8].

In comparison with thin films, nanostructure-based RRAM devices offer structural advantages that are expected to improve the uniformity of memory devices [9,10]. The distinct geometry of nanorods (NRs) can control the distribution of oxygen vacancies due to the formation of straight and extensible conducting filaments along the direction of each NRs, thereby improving the uniformity of memory devices [9,11]. In such devices, nanostructure materials, such as GZO NRs [12], NiO/Pt nanowires [13], and Ni–NiO core–shell nanowires [14], have been reported to show resistive switching characteristics with filament formation and current flow. Although these nanostructure materials show resistive switching behavior, they demand the forming process and their ON/OFF ratio is not good. In addition, nanostructure materials are challenging to grow.

In this work, bipolar resistive switching and forming-free MZN NR memory devices were prepared by hydrothermal method. This method has attracted considerable attention due to its simple procedure, low cost, low temperature, high yield, and controllable process [15,16,17]. MZN NRs can control oxygen vacancy distribution because injected electrons will be trapped by uniform ionized oxygen vacancies distributed on the MZN NR sidewall. MZN NR memory devices can reduce the coefficient of variation (CV) of ILRS/IHRS from 50%/90% to 30%/50%. In addition, MZN NR-based RRAM device exhibits good retention up to 10^4^ s and high ON/OFF ratio of more than 10^6^.

## 2. Results

**Figure 1:**
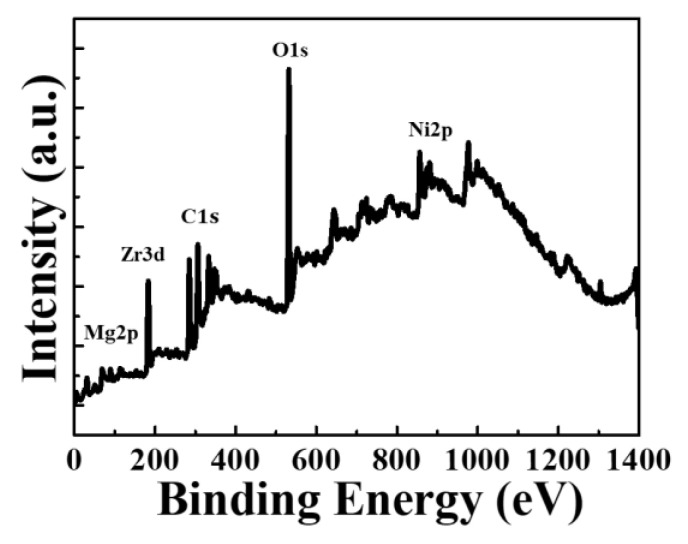
XPS spectra of O 1s, Ni 2p3, Mg 2p, Zr 3d, and C 1s.

Figure 1 shows the X-ray photoelectron spectroscopy (XPS) spectra of the MZN thin film. The atomic percentage of elements magnesium, zirconate, nickel, and oxygen in MZN thin films verified by XP spectra is 9.2%, 21.2%, 21.2%, and 69.6%, respectively.

**Figure 2:**
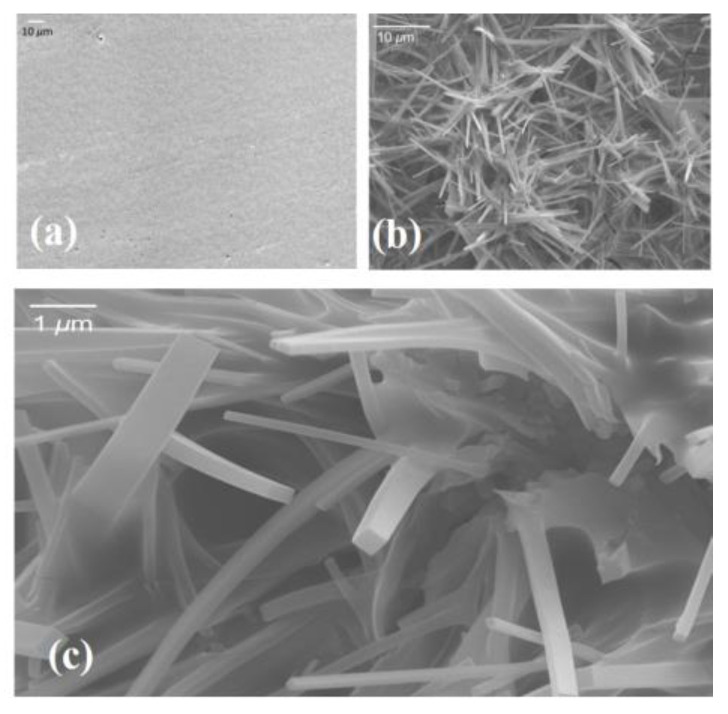
(**a**) SEM image of the surface morphologies of MZN thin film. (**b**) Plan-view SEM data of MZN NR nucleation layer that was formed in situ on an ITO surface from the decomposition of the MZN precursor at 60 °C. MZN NRs were grown for 23 h. (**c**) High-resolution SEM image of MZN NRs.

Scanning electron microscope (SEM) image shows the formation of MZN NRs on the MZN/ITO structure. Figure 2a shows the smooth surface morphology of the MZN thin film. Scattered MZN NRs were clearly observed on the surface of the MZN thin film soaked for 23 h (Figure 2b). The shape of the MZN NR is cuboid with width of about 200 (Figure 2c).

**Figure 3:**
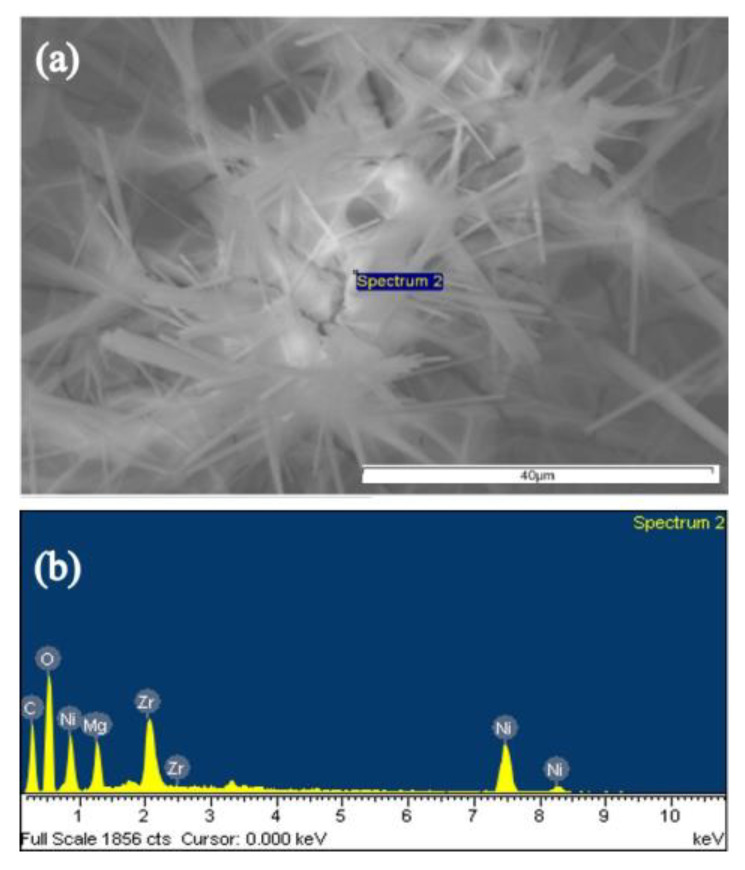
EDS analysis of MZN NR-based thin film (**a**) testing location and (**b**) chemical composition.

Metal chemical components on MZN NR thin film were determined by energy dispersive spectroscopy (EDS, Figure 3). Figure 3a shows the testing location in the MZN NR thin film. Figure 3b and Table 1 show that the chemical composition of MZN NR thin film includes Mg = 3.15%, Zr = 2.15%, Ni = 5.38%, C = 49.13%, and O = 40.2%.

**Figure 4:**
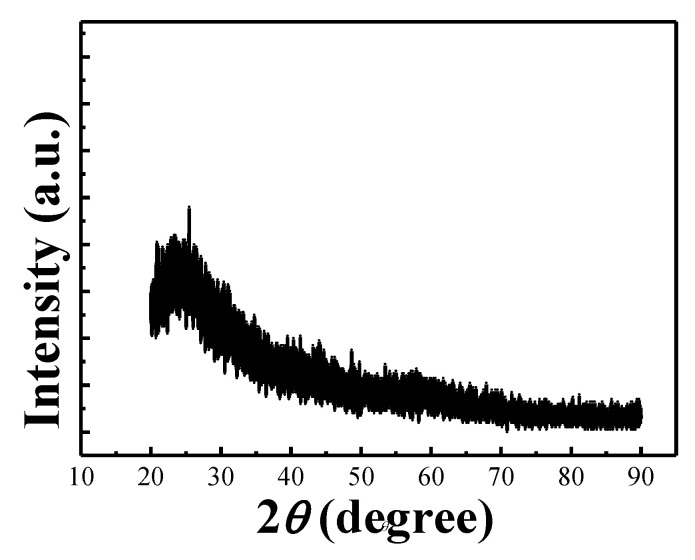
XRD pattern of MZN NR/glass thin films.

The X-ray diffraction analysis of the MZN NR thin film/glass is depicted in Figure 4. No obvious peak was observed in the XRD spectrum of MZN NR/glass. The MZN NR thin film prepared in this experiment is amorphous phase. Compared with polycrystalline phase, amorphous phase is preferred for dielectric layer material because the former may have a high-grain boundary leakage current and lead to a rough film surface [18].

**Figure 5:**
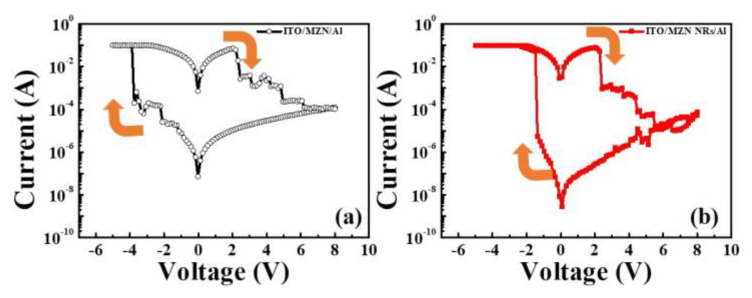
Typical *I–V* curves of: (**a**) Al/MZN thin film/ITO structure; and (**b**) Al/MZN NR/ITO structure.

The bipolar resistance switching characteristics of the fabricated Al/MZN NR/ITO RRAM were compared with the device without NRs (Figure 5). Low-temperature hydrothermal method can offer sufficient oxygen vacancy [19]. For the NR-based RRAM, the set voltage is −1.3 V and the reset voltage is 2.2 V, which is smaller than those in devices without NRs. By sweeping a negative bias from zero to over the set voltage, the device switched from the high resistance state (HRS) to the low resistance state (LRS), called “the set process”. When a positive reset voltage was applied, the device could return to HRS, which is the “reset process”. Compared with MZN-based RRAM, the ON/OFF ratio (read at 0.1 V) of the Al/MZN NR/ITO device could be increased from 10^4^ to 10^6^.

**Figure 6:**
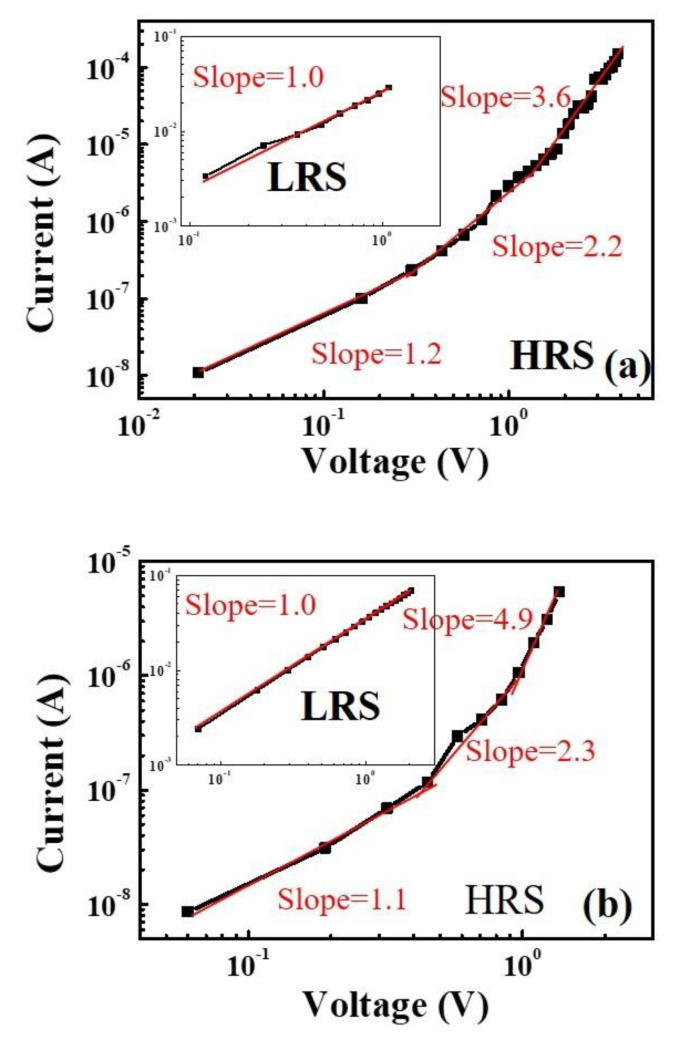
Log *I*–log *V* curves for the positive voltage regions and the negative voltage regions of: (**a**) Al/MZN/ITO device; and (**b**) Al/MZN NR/ITO device. The inset image shows the Log *IV* curves for the LRS.

Figure 6 shows the logarithmic plots of *I–V* curves for positive and negative voltage sweep regions of (Figure 6a) Al/MZN/ITO and (Figure 6b) Al/MZN NR/ITO devices, respectively. The log *I*−log *V* curves in the HRS of Al/MZN/ITO and Al/MZN NRs/ITO devices at low voltage have a linear region with a slope of 1.01, thus implying the ohmic conduction behavior of both devices. In the high-voltage region, these slopes are related with Child’s law (I α V^2^), presenting a space charge limited conduction (SCLC). RRAM devices without and with NRs were dominated by space-charge-limited current (SCLC) in the HRS [20]. By contrast, the relationship between log *I* and log *V* in the LRS showed linear dependence, corresponding to ohmic conduction behavior. This finding implies that resistive switching mechanisms could be considered as formation and rupture of conducting filament paths.

**Figure 7:**
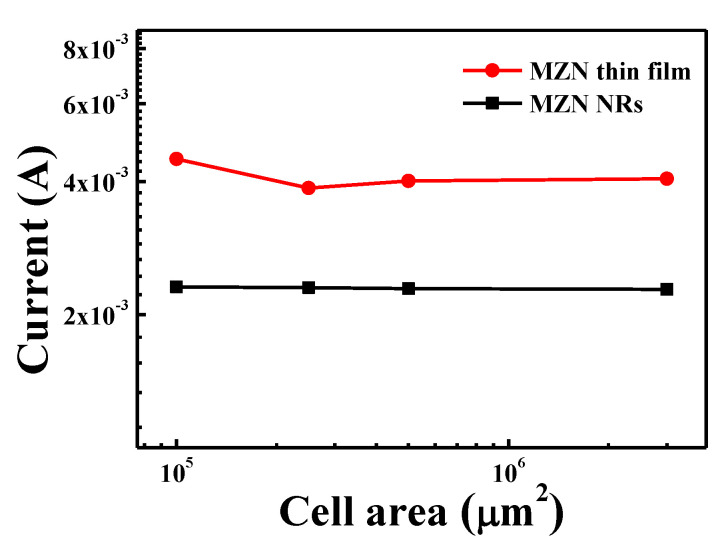
LRS currents versus cell areas of Al/MZN/ITO and Al/MZN NR/ITO devices.

Figure 7 shows the plots the scaling trend of the LRS current versus the cell area of Al/MZN/ITO and Al/MZN NR/ITO devices. The LRS current showed a cell size-independent behavior. LRS current as a filamentary conduction current only had a slight dependence on cell area [21]. This result indicates that resistive switching behavior was related to and occurred in conductive filaments.

**Figure 8:**
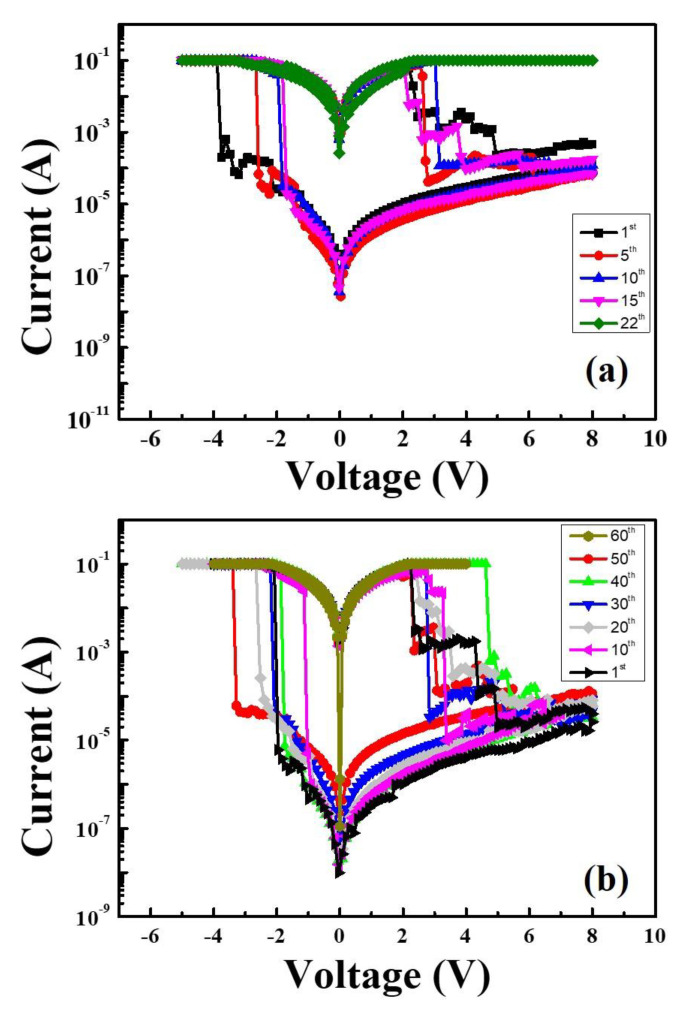
Switching cycles of: (**a**) Al/MZN/ITO device; and (**b**) Al/MZN NR/ITO device.

Figure 8 shows the endurance switching cycles of (Figure 8a) Al/MZN/ITO and (Figure 8b) Al/MZN NR/ITO structures, respectively, under DC voltage sweep. Figure 8a shows the Al/MZN/ITO structure can maintain the ON/OFF ratio of more than 10^4^ read at 0.1 V after 20 continuous DC voltage switching cycles. Figure 8b shows the switching endurance of Al/MZN NR/ITO memory cell over 50 switching cycles with ON/OFF ratio was approximately 10^5^ [22].

**Figure 9:**
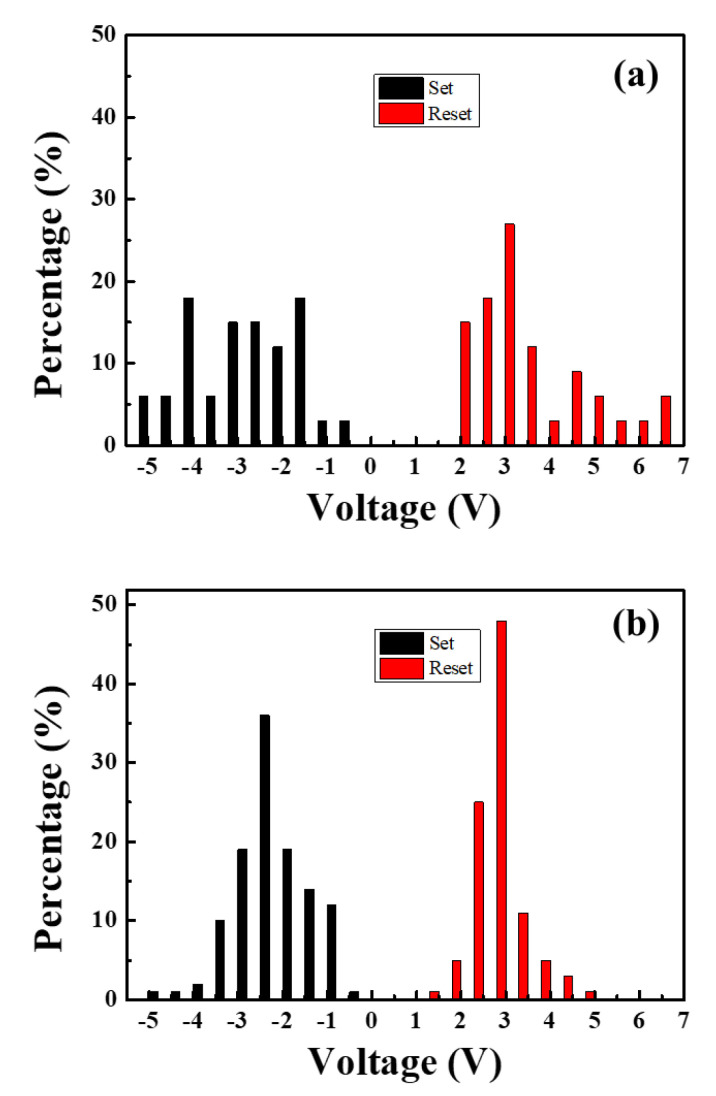
Set and reset voltage distributions of: (**a**) Al/MZN/ITO/glass device; and (**b**) Al/MZN NR/ITO/glass device.

Figure 9 shows the V*_set_*/V*_reset_* distributions of (Figure 9a) Al/MZN/ITO and (Figure 9b) Al/MZN NR/ITO devices. This uniformity is better than that of Al/MZN/ITO devices (Figure 9a), whose set voltage was distributed from −5 to −0.5 V and their reset voltage was distributed from 2 to 7 V. Figure 9b shows the V*_set_* of Al/MZN NR/ITO devices was distributed within −5 to −0.5 V, and V*_reset_* was distributed within 1.5–5 V when the devices switched from the ON to the OFF state.

**Figure 10:**
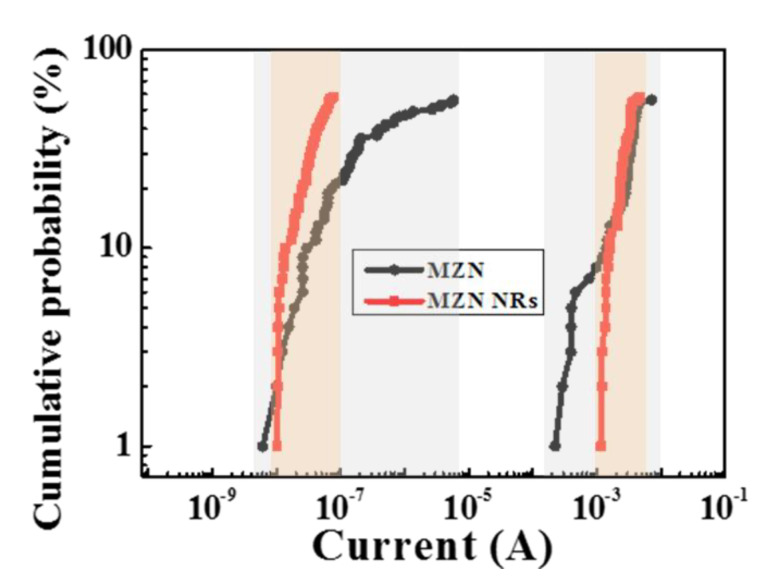
Current statistical distributions of: (**a**) Al/MZN/ITO device; and (**b**) Al/MZN NR/ITO device.

Figure 10a,b presents the current distributions of Al/MZN/ITO and Al/MZN NR/ITO devices, respectively. CV was used to evaluate the distribution. The CVs of the HRS and LRS currents of Al/MZN NR/ITO devices were 96% and 52%, respectively. The CVs of HRS and LRS currents in the MZN-based RRAM devices were 50% and 30%, respectively [23]. Furthermore, LRS and HRS were distributed more uniformly after adding NRs, and the ON/OFF ratio was maintained at approximately 10^5^. The MZN NRs can concentrate oxygen vacancies on the surface of MZN NR, instead of distributing oxygen vacancies in the smooth MZN thin film. Therefore, MZN NRs can improve the uniformity of conductive filaments and thus reduce the CV.

**Figure 11:**
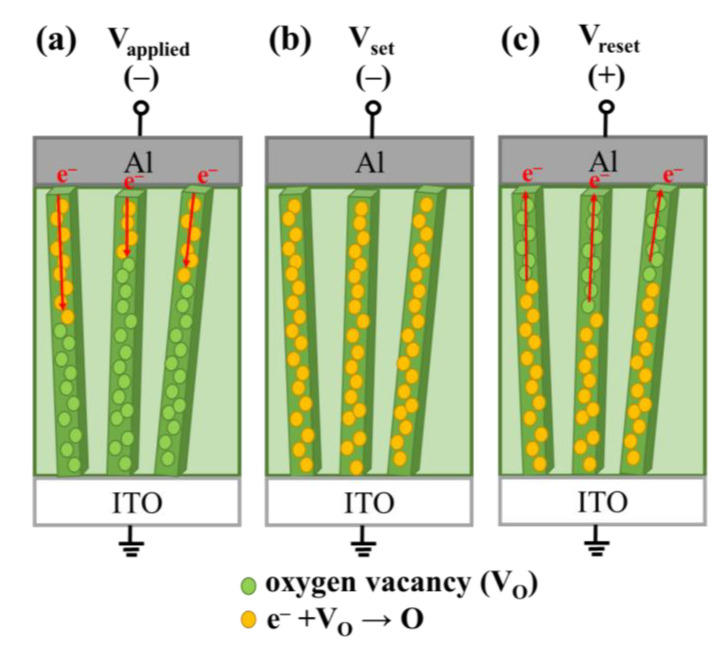
Schematic of the mechanisms of MZN NRs in terms of: (**a**) distribution of oxygen vacancies on the NR sidewall; (**b**) conductive filaments; and (**c**) rupture of conductive filaments.

In this study, we supposed that ionized oxygen vacancies charge and discharge electrons to achieve cycles of the formation and rupture of conductive filaments on the surface of MZN NRs according to the conduction mechanism [8,9,24,25]. Figure 11a shows that the injected electrons were trapped on the NR sidewall with uniform distribution of ionized oxygen vacancies. As shown in Figure 11b, the injected electrons fully occupied the trap level when the applied voltage was gradually increased to the set voltage, and subsequent electrons could transport to the ITO without being trapped. Conductive filaments are bridged on the NR sidewall. Figure 11c shows that, under negative bias, the captured electrons were released to the Al due to the negative electric field, resulting in an increase in the resistance. This phenomenon may explain the rupture of conductive filaments and the reset process.

**Figure 12:**
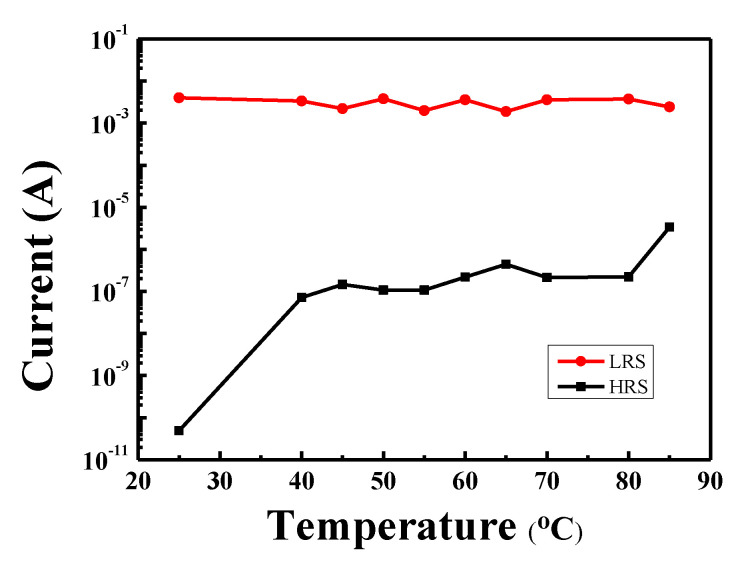
The typical resistive switching current–voltage curve of Al/MZN NR/ITO RRAM device at different environmental temperatures.

Figure 12 shows the ON/OFF ratio variation in Al/MZN NR/ITO devices with increasing temperature, in which the current was measured at 0.1 V. The ON/OFF ratio measured at room temperature was approximately 10^7^, and the HRS current was 4.9 × 10^−11^ A. When the temperature was increased from 40 to 85 °C, the HRS current increased to around 10^−7^ A, and the ON/OFF ratio decreased to 10^4^ and kept the memory window without significant degradation. The result demonstrates the steady resistive switching operation of Al/MZN NR/ITO devices with temperature variation.

**Figure 13:**
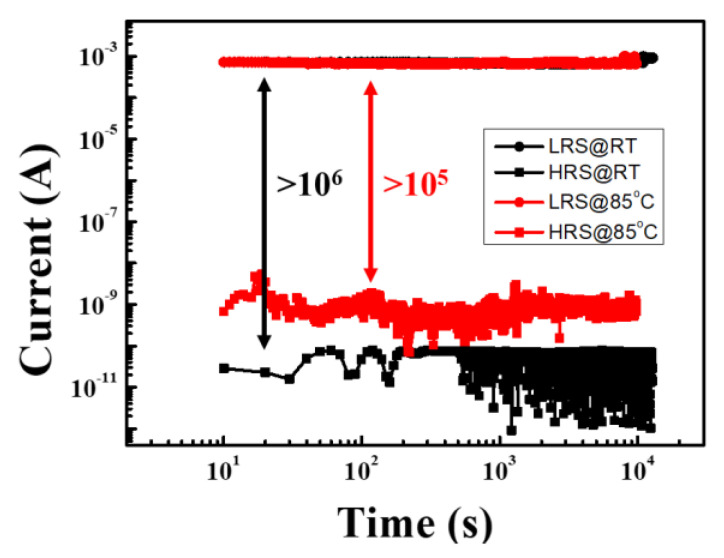
Data retention characteristics of Al/MZN NR/ITO RRAM devices measured at 0.1 V.

Figure 13 presents the retention time of Al/MZN NR/ITO RRAM devices measured at room temperature and at 85 °C. The ON/OFF ratio was maintained to at least 10^6^ and 10^5^ at room temperature and at 85 °C, respectively. The HRS and LRS were read at 0.1 V for over 10^4^ s. Hence, good retention of data could be obtained, clearly revealing the nonvolatile characteristics of RRAM devices.

## 3. Materials and Methods

**Figure 14:**
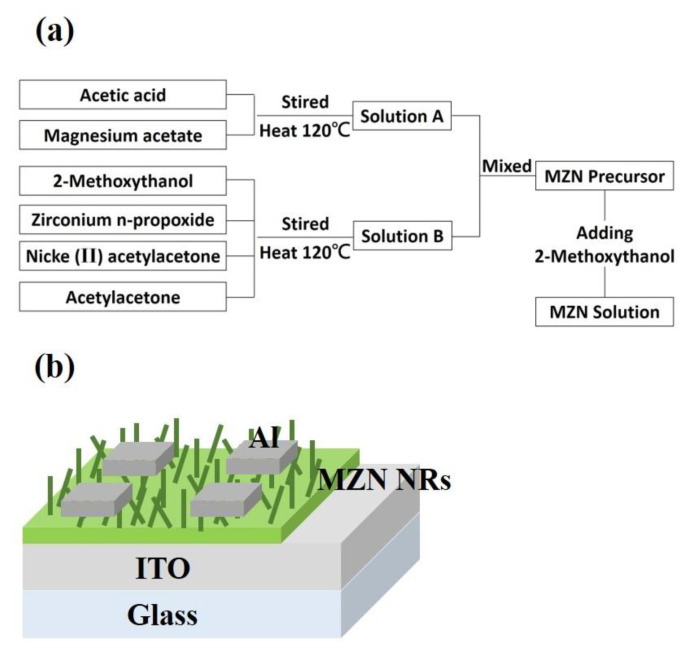
(**a**) Flow chart for preparation of 0.5 M MZN solution; and (**b**) schematic of Al/MZN NR/ITO device and set up for measurement.

MZN solution (0.5 M) was prepared as follows. Magnesium acetate, zirconium n-propoxide, and nickel II acetylacetone were synthesized according to the flow chart shown in Figure 14a. An appropriate amount of magnesium acetate was added to glacial acetic acid to obtain Solution A. Zirconium n-propoxide was dissolved into 2-methoxythanol and added with nickel (II) acetylacetone and acetylacetone to produce Solution B. Solutions A and B were mixed. MZN NR film was synthesized through hydrothermal method. The MZN thin film was deposited by spin coating onto the ITO/glass substrate to act as a NR nucleation layer and baked at 100 °C for 10 min. The MZN NR film formed an MZN NR nucleation layer in 1 M MZN solution at 60 °C for 23 h. After the growth of the MZN NRs, Al top electrodes were deposited with 3 mm^2^ patterned shadow mask by using direct current (DC) magnetron sputtering. Figure 14b shows the schematic of the MZN NR-based RRAM.

## 4. Conclusions

MZN NRs were grown on ITO/glass substrate through hydrothermal method. Compared with its counterpart MTN thin film, MZN NR RRAM can significantly enhance the uniformity and the ON/OFF ratio from 10^4^ to 10^6^ without any forming processes. In addition, the CVs of MZN NR memory devices were 29% for ILRS and 46% for IHRS, which are lower than those of MZN film memory device (52% for ILRS and 97% for IHRS). This result indicates that MZN NRs improved device uniformity. The electrical conduction mechanism exhibited a trap-controlled space-charge limited current. Ionized oxygen vacancies distributed on the surface of MZN NRs may lead to more uniform switching distribution. When applying the set voltage, the electrons were trapped on the NR sidewall and conductive filaments were formed, which set the device to LRS. When applying the reset voltage, the captured electrons were released and the device was reset to HRS.

## Figures and Tables

**Table 1:** Atomic percentage of MZN NR-based thin films.

Element	Mg	Zr	Ni	C	O	Totals
Atomic (%)	3.15	2.15	5.38	49.13	40.20	100%
